# Computed tomography imaging of macrophage phagocytic activity in abdominal aortic aneurysm

**DOI:** 10.7150/thno.55106

**Published:** 2021-04-03

**Authors:** Jakub Toczek, Parnaz Boodagh, Nowshin Sanzida, Mean Ghim, Mani Salarian, Kiran Gona, Gunjan Kukreja, Saranya Rajendran, Linyan Wei, Jinah Han, Jiasheng Zhang, Jae-Joon Jung, Morven Graham, Xinran Liu, Mehran M. Sadeghi

**Affiliations:** 1 Cardiovascular Molecular Imaging Laboratory, Section of Cardiovascular Medicine and Yale Cardiovascular Research Center, Yale University School of Medicine, New Haven, CT (USA).; 2 Veterans Affairs Connecticut Healthcare System, West Haven, CT (USA).; 3 CCMI Electron Microscopy Core Facility, Yale University School of Medicine, New Haven, CT (USA).

**Keywords:** molecular imaging, computed tomography, nanoparticles, inflammation, abdominal aortic aneurysm

## Abstract

Inflammation plays a major role in the pathogenesis of several vascular pathologies, including abdominal aortic aneurysm (AAA). Evaluating the role of inflammation in AAA pathobiology and potentially outcome *in vivo* requires non-invasive tools for high-resolution imaging. We investigated the feasibility of X-ray computed tomography (CT) imaging of phagocytic activity using nanoparticle contrast agents to predict AAA outcome.

**Methods:** Uptake of several nanoparticle CT contrast agents was evaluated in a macrophage cell line. The most promising agent, Exitron nano 12000, was further characterized *in vitro* and used for subsequent *in vivo* testing. AAA was induced in *Apoe*^-/-^ mice through angiotensin II (Ang II) infusion for up to 4 weeks. Nanoparticle biodistribution and uptake in AAA were evaluated by CT imaging in Ang II-infused *Apoe*^-/-^ mice. After imaging, the aortic tissue was harvested and used from morphometry, transmission electron microscopy and gene expression analysis. A group of Ang II-infused *Apoe*^-/-^ mice underwent nanoparticle-enhanced CT imaging within the first week of Ang II infusion, and their survival and aortic external diameter were evaluated at 4 weeks to address the value of vessel wall CT enhancement in predicting AAA outcome.

**Results:** Exitron nano 12000 showed specific uptake in macrophages *in vitro*. Nanoparticle accumulation was observed by CT imaging in tissues rich in mononuclear phagocytes. Aortic wall enhancement was detectable on delayed CT images following nanoparticle administration and correlated with vessel wall CD68 expression. Transmission electron microscopy ascertained the presence of nanoparticles in AAA adventitial macrophages. Nanoparticle-induced CT enhancement on images obtained within one week of AAA induction was predictive of AAA outcome at 4 weeks.

**Conclusions:** By establishing the feasibility of CT-based molecular imaging of phagocytic activity in AAA, this study links the inflammatory signal on early time point images to AAA evolution. This readily available technology overcomes an important barrier to cross-sectional, longitudinal and outcome studies, not only in AAA, but also in other cardiovascular pathologies and facilitates the evaluation of modulatory interventions, and ultimately upon clinical translation, patient management.

## Introduction

Inflammation is a key feature in chronic cardiovascular diseases such as abdominal aortic aneurysm (AAA) 1, 2. The prominent role of inflammation in the pathogenesis of AAA and other vascular pathologies is now well recognized 3. Inflammatory cells, including macrophages, and the soluble factors they secrete promote extracellular matrix remodeling and loss of media smooth muscle cells (SMCs), the main pathologic processes in AAA development 4. In the absence of appropriate imaging tools, studies aimed at evaluating the role of inflammation in vascular pathology have relied on leukocyte depletion or genetic interventions to reduce leukocyte function and chemotaxis 3, 5, 6. While powerful, these interventional approaches affect the natural history of the disease and as such, are of limited value in linking vessel wall inflammation to future outcomes. Tools for non-invasive evaluation of vessel wall inflammation *in vivo* could address this gap, advance vascular biology research and help address unmet needs in drug development and potentially, patient management 7.

Detection of inflammation on a systemic level can be achieved using blood biomarkers, such as C-reactive protein or white blood cell count 8, 9. However, systemic biomarkers can be influenced by unrelated inflammatory foci. This lack of specificity can be overcome by imaging the inflammatory process in the vessel wall. Accordingly, several molecular imaging approaches for noninvasive detection of aortic wall inflammation have been introduced and evaluated in preclinical models of AAA and human subjects 10. Most of these are based on nuclear imaging technologies, that while highly sensitive, are of limited spatial resolution and may lack specificity 11. An alternative approach by magnetic resonance imaging (MRI) uses ultrasmall superparamagnetic iron oxide particles (USPIO) to target the phagocytic activity of inflammatory cells 12-14. This approach was shown to be predictive of AAA outcome, although not independent of known clinical risk factors 14. However, USPIO-enhanced MRI image acquisition and analysis, especially in small animals, can be challenging. In addition, this technique is not truly quantitative.

X-ray computed tomography (CT) is a readily available technology that provides high spatial resolution, and fast image acquisition at low cost. This has led to extensive utilization of CT as the preferred imaging modality for a broad range of applications 15. However, the poor soft tissue contrast of CT mandates the use of X-ray attenuating contrast media for a number of applications. In the last two decades, a variety of nanoparticle contrast agents for X-ray imaging have been developed 16, 17. These nanoparticles can enter cells by phagocytosis, which takes place mainly in professional phagocytes, cells from the mononuclear phagocytic system (MPS), such as macrophages 18, 19. We hypothesized that phagocytic uptake of CT contrast nanoparticles may be leveraged to detect vessel wall inflammation *in vivo* by CT. To test this hypothesis, we used AAA as a prototypic model of vessel wall inflammation to demonstrate the feasibility of CT imaging of vessel wall inflammation *in vivo* and address its functional significance in predicting AAA outcome in murine models.

## Results

### Nanoparticle contrast agent selection

**Figure 1:**
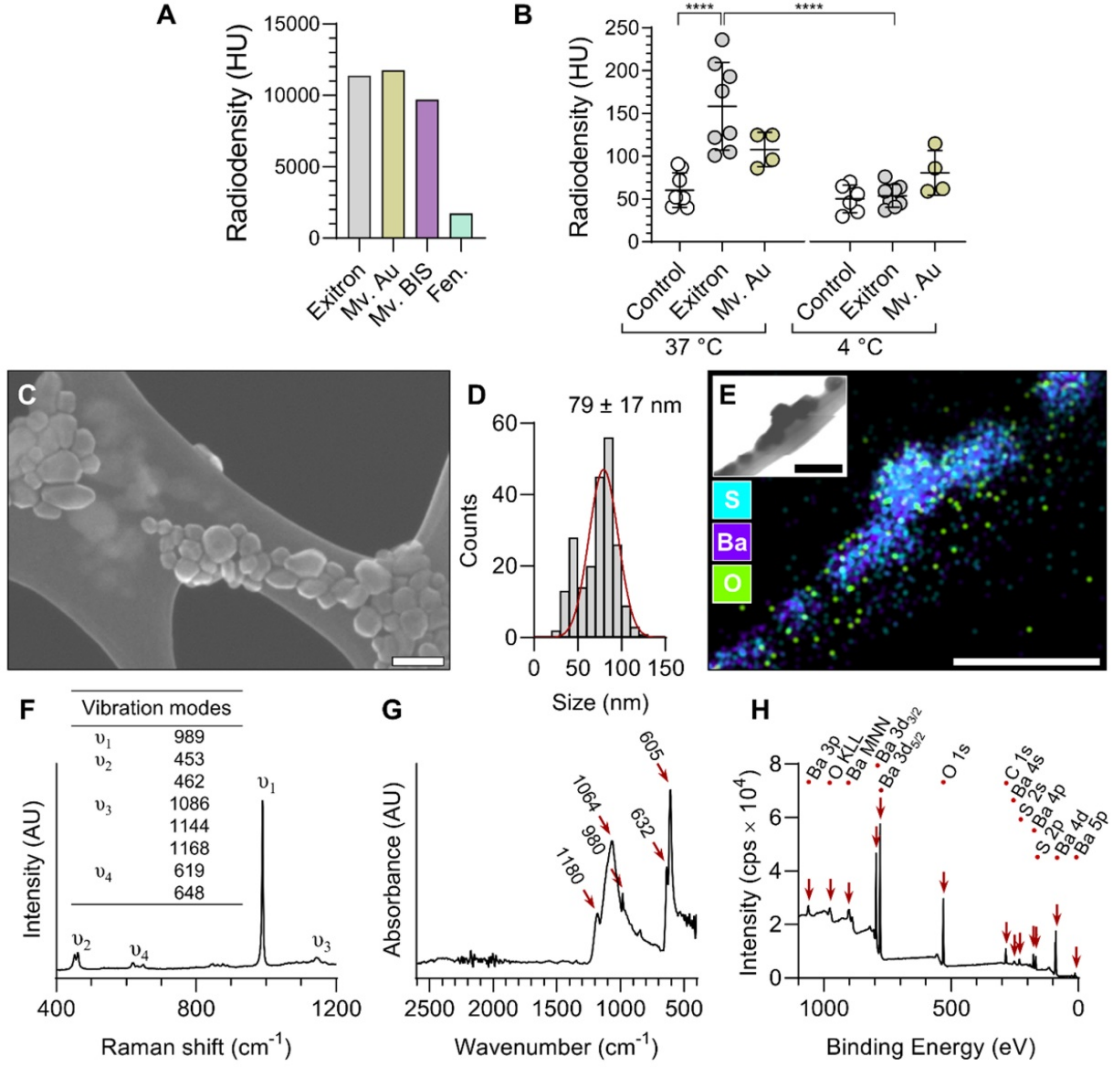
*In vitro* evaluation and characterization of nanoparticle CT contrast agents. (A) Radiodensity measurement of the contrast agent stock solutions. (B) CT measurement of nanoparticle contrast agent uptake by RAW 264.7 macrophages at 37 and 4 °C (n = 4-8). Statistical significance was assessed by one-way ANOVA with Tukey's correction. ****P < 0.0001. (C) A representative scanning electron microscopy image and (D) corresponding size distribution of Exitron nanoparticles. (E) Energy dispersive X-Ray spectroscopy elemental mapping of Exitron. Inset shows the corresponding transmission electron microscopy image. (F) Raman spectrum of Exitron nanoparticles. (G) Fourier-transform infrared absorbance spectrum of Exitron nanoparticles. (H) X-ray photoelectron spectroscopy spectrum of Exitron nanoparticle surface elements. Exitron: Exitron nano 12000; Mv. Au: Mvivo Au; Mv. BIS: Mvivo BIS; Fen.: Fenestra LC; HU: Hounsfield units. Scale bar: 100 nm.

Four formulations of nanoparticle CT contrast agents with distinct structural compositions, Exitron nano 12000 (Exitron), Mvivo Au, Mvivo BIS and Fenestra LC, were evaluated for their uptake by RAW 264.7 macrophage cells. The radiodensity of each stock solution was determined by CT imaging (Figure 1A) and the cells were exposed to dilutions of contrast agents yielding a similar radiodensity. Due to its poor solubility in aqueous solution (culture media or PBS), Mvivo BIS was excluded from *in vitro* uptake analysis (Figure S1). In addition, the tested concentration of Fenestra LC resulted in considerable cell toxicity. As a result, cellular uptake of nanoparticles could only be assessed for Exitron and Mvivo Au. Incubation with Exitron significantly increased the radiodensity of the cell mass at 37 °C (*P* < 0.0001 vs. control condition), but not at 4 °C (*P* = ns vs. control condition), indicating that nanoparticle internalization occurred through an active process (Figure 1B). No statistically significant change in radiodensity was observed upon incubation of the cells with Mvivo Au (Figure 1B). Accordingly, we selected Exitron as the lead nanoparticle CT contrast agent for further evaluation. To investigate the effect of Exitron on macrophage polarization and the effect of macrophage polarization on Exitron uptake, RAW 264.7 macrophages cells were polarized with lipopolysaccharide and interferon gamma (LPS+INFγ) or interleukin-4 (IL-4). As expected, the classical polarization marker *Nos2* and alternative polarization marker *Mrc1* were upregulated upon stimulation with LPS+INFγ and IL-4, respectively (Figure S2A). In parallel, exposure to Exitron for up to 48 h did not increase *Nos2* or *Mrc1* expression in RAW 264.7 cells (Figure S2B). Evaluation of Exitron uptake by polarized macrophages showed that Exitron uptake is abrogated in classically polarized macrophages (Figure S3A). Finally, in addition to uptake by professional phagocytes, Exitron uptake was also observed in non-professional phagocyte endothelial and smooth muscle cell lines *in vitro* (Figure S3B, C).

### Characterization of Exitron

Exitron nano 12000 is a commercial nanoparticle CT contrast agent with limited public information on its composition and characteristics. Therefore, we performed a series of analytical studies to characterize this agent. Scanning electron microscopy (SEM) showed some heterogeneity in the shape and size (79 ± 17 nm) of the particles (Figure 1C-D). Energy-dispersive X-ray spectroscopy (EDS) elemental mapping showed the presence of barium, sulfur and oxygen in the particles (Figure 1E), and Raman spectroscopy analysis revealed a strong ν_1_ band corresponding to the symmetric stretching vibration of sulfate tetrahedra and other characteristic bands matching the barium sulfate (BaSO_4_) spectrum (Figure 1F). Fourier-transform infrared spectroscopy (FTIR) analysis yielded concordant results, with a spectrum matching the profile of BaSO_4_ (Figure 1G). Surface-sensitive X-ray photoelectron spectroscopy (XPS) confirmed the presence of barium, sulfur, oxygen and carbon atoms within the surface layer of nanoparticles, and identified doublet peaks of Ba 3d_5/2_ and 3d_3/2_ with spin-orbit splitting of 15 eV, confirming the Ba^2+^ ionic state (Figure 1H). Finally, the zeta potential of nanoparticles dispersed in water was measured to be -19.2 ± 1.3 mV.

### Exitron biodistribution in C57Bl/6J mice

**Figure 2:**
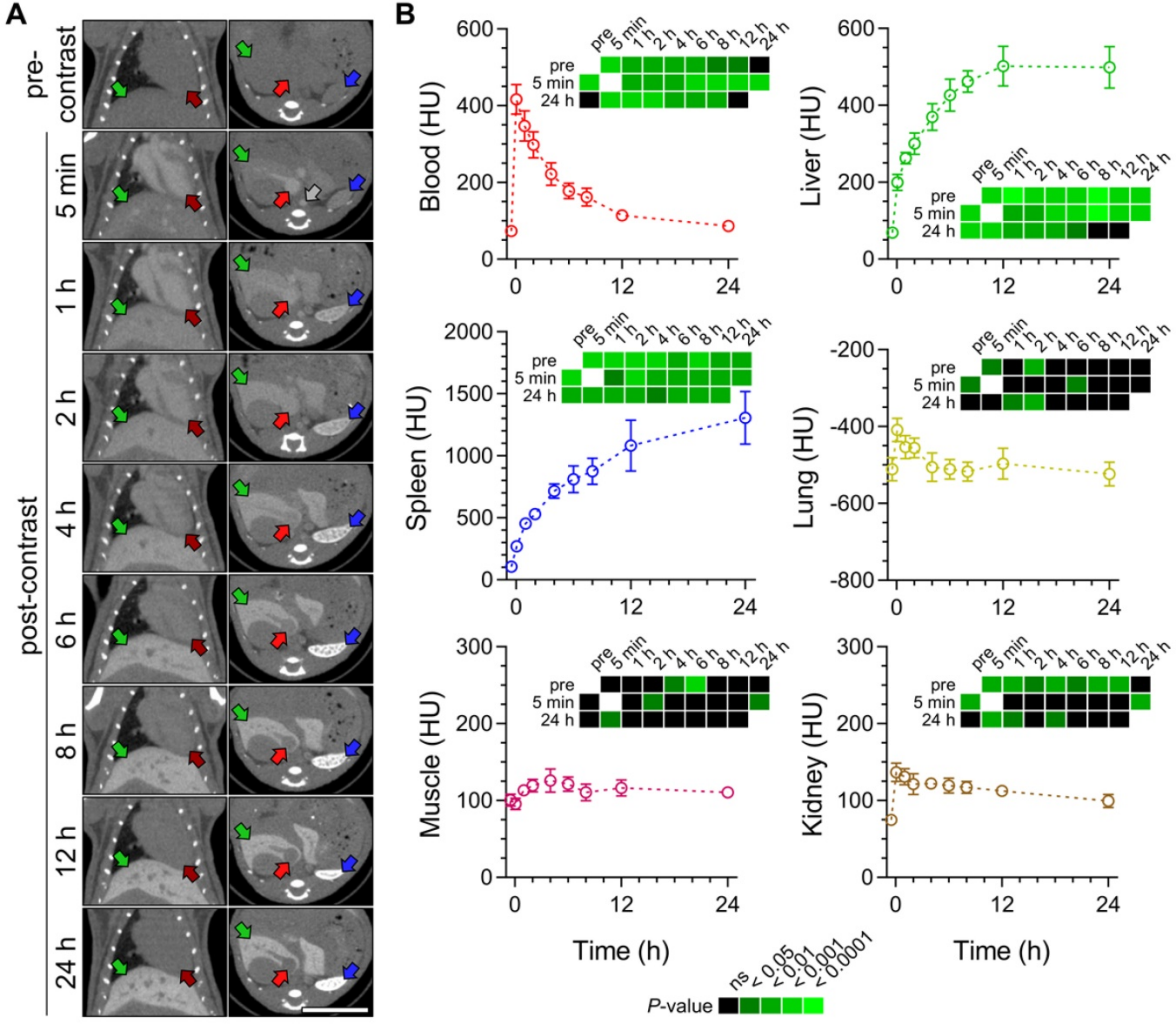
Exitron nano 12000 biodistribution in C57Bl/6J mice. (**A**) Examples of CT images acquired before and over 24 h after the injection of Exitron. Grey arrow: supra-renal abdominal aorta; dark red arrows: left ventricular cavity; red arrows: inferior vena cava; green arrows: liver; blue arrows: spleen. Scale bar: 1 cm. CT scale: -750 to 1250 Hounsfield Units (HU). (**B**) Quantification of mean radiodensity of different tissues over time (n = 5). Statistical significance was assessed by one-way ANOVA with Geisser-Greenhouse and Tukey's corrections; group comparisons are shown on heatmaps subpanels.

As a prelude to *in vivo* imaging studies in AAA, we evaluated the temporal changes in Exitron biodistribution in C57Bl/6J mice by acquiring serial CT images *in vivo* following intravenous administration of the contrast agent [50 μL per 25 g of body weight (BW)]). The CT images showed an initial increase in blood, and to a lesser extent, other tissues' radiodensity. The radiodensity of the blood and other evaluated tissues slowly decreased over the following 24 hours, except for the liver and spleen, where a progressive increase in tissue radiodensity was detected consistent with nanoparticle accumulation in the MPS cells (Figure 2).

### Exitron uptake in AAA

**Figure 3:**
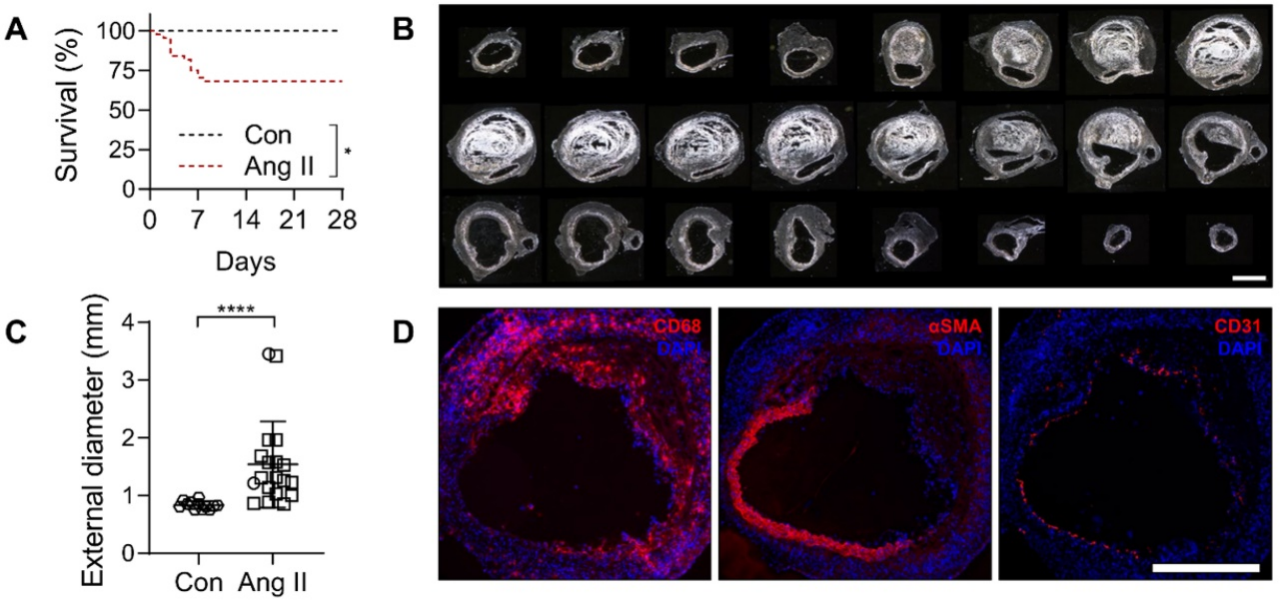
Characterization of AAA in Ang II-infused *Apoe^-/-^* mice. (**A**) Kaplan-Meier survival curves of Ang II-infused (n = 44) and saline-infused (Con, n = 12) *Apoe^-/-^* mice. The curves were compared using Log-rank (Mantel-Cox) test. * *P* < 0.05. (**B**) Darkfield images of serial sections of abdominal aorta of a representative Ang II-infused mouse with AAA, starting below the diaphragm and ending in the infra-renal aorta. Scale bar: 1 mm. (**C**) Maximal external diameter of supra-renal aorta after 4 weeks of saline (n = 12) or Ang II infusion in *Apoe^-/-^* mice (n = 19); the difference between mean values was compared using Mann-Whitney U test. ****P < 0.0001. (**D**) Representative immunofluorescence staining of macrophages (CD68), smooth muscle cells (αSMA) and endothelial cells (CD31) in AAA. Nuclei are stained in blue with DAPI. Scale bar: 500 µm.

To induce AAA, a group of male *Apoe^-/-^* mice (n = 44) were infused with angiotensin (Ang) II for a period of 4 weeks. Of these, 32% (14/44) died of rupture within the first 8 days of Ang II infusion. No rupture was observed in saline-infused, control *Apoe^-/-^* mice (n = 12) (Figure 3A). The maximal external diameter of suprarenal abdominal aorta in animals that survived the 4 weeks of Ang II infusion (1.54 ± 0.66 mm, range 0.85 to 3.45 mm) was significantly larger than the corresponding aortic diameter in saline-infused animals (0.83 ± 0.07 mm, range 0.75 to 0.97 mm, *P* < 0.0001) (Figure 3B-C). The presence of CD68-positive macrophages, loss of α-smooth muscle actin-positive SMC, and to a lesser extent, loss of CD31-positive endothelial cells was readily detectable by Immunostaining in AAA (Figure 3D).

**Figure 4:**
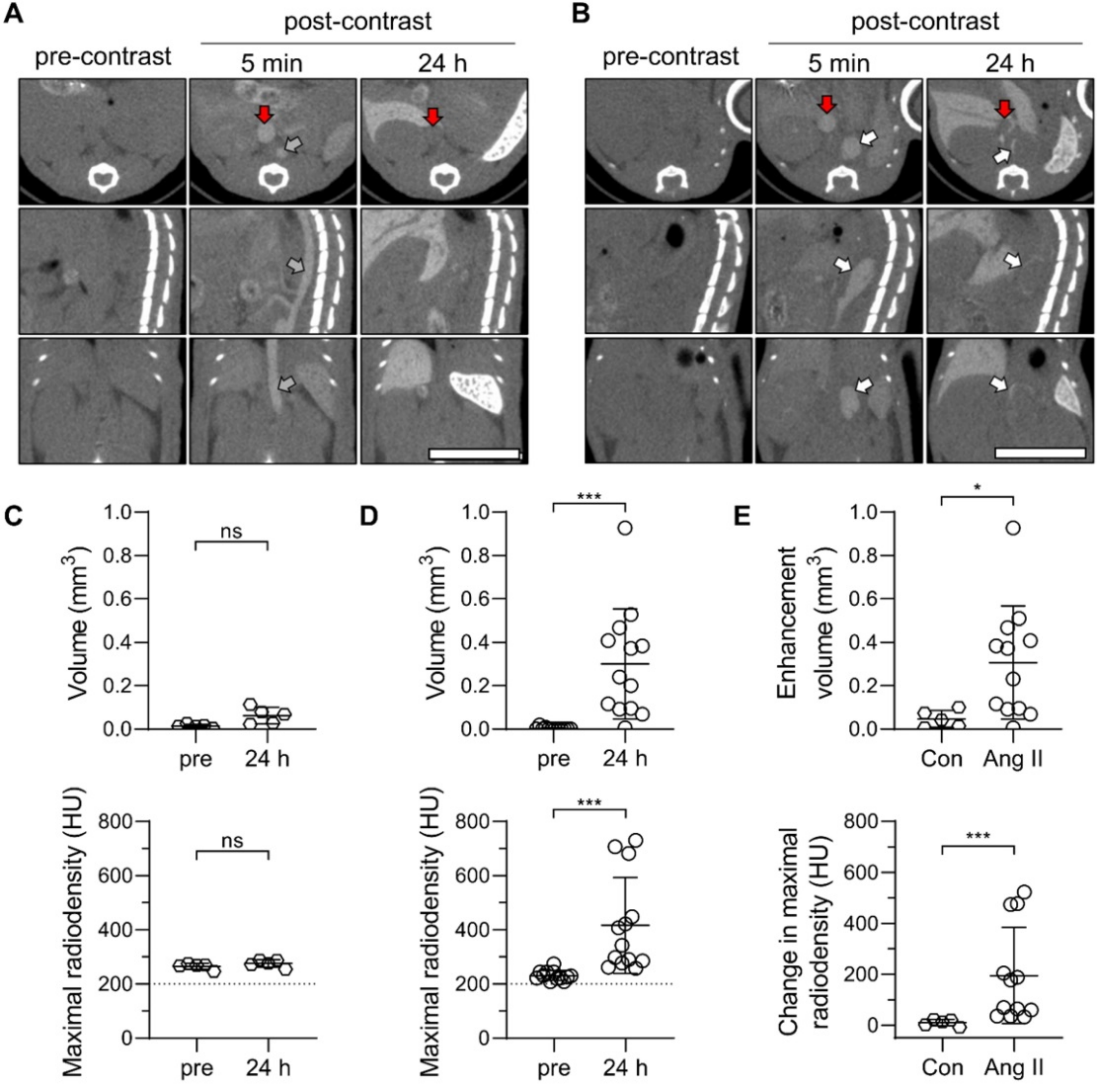
Exitron nano 12000 uptake in Ang II-induced AAA in *Apoe^-/^*^-^ mice. (**A**, **B**) Representative CT images of C57Bl/6J mice (Con, n = 5, **A**) and Ang II-infused *Apoe^-/-^* mice with AAA (n = 13, **B**) prior to (pre-contrast), and 5 min and 24 h after the administration of Exitron nano 12000 (post-contrast). Grey arrow: abdominal aorta, white arrows: AAA; red arrows: inferior vena cava. Scale bar: 1 cm. CT scale: -750 to 1250 HU. (**C**, **D**) Threshold-based quantification of the supra-renal abdominal aorta Exitron signal in Con (**C**) and Ang II-infused mice (**D**) expressed as enhancement volume (upper panels) and maximal radiodensity (lower panels); statistical significance was assessed by paired t-test (panels in **C**) or Wilcoxon's rank test (panels in **D**). (**E**) Signal enhancement between pre-injection and 24 h post-injection images quantified as enhancement volume (upper panel) and change in maximal radiodensity (lower panel); statistical significance was assessed using unpaired t-test (upper panel) and Mann-Whitney U test (lower panel).

Next, a group of Ang II-infused and control animals underwent serial CT imaging before, and at 5 minutes and 24 h after intravenous administration of Exitron (50 μL/25 g of BW). The slow blood clearance of the contrast agent facilitated the visualization of major blood vessels, including the aorta and inferior vena cava on images acquired at 5 minutes after contrast administration (Figure 4A-B). In animals with AAA, suprarenal aortic dilatation, associated in some cases with a false lumen, was readily detectable on these images (Figure 4B). Blood pool clearance on images acquired after 24 hours highlighted the opacification of the AAA wall, indicative of nanoparticle retention in this area (Figure 4B). Threshold-based quantification of the nanoparticle signal confirmed the absence of abdominal aortic wall opacification in control animals (Figure 4C), while there was a significant increase in aortic wall opacification volume and maximal radiodensity post-contrast in animals with AAA (*P* < 0.001 vs. pre-injection images for both, Figure 4D). Accordingly, contrast-induced aortic wall enhancement on CT images was more pronounced in animals with AAA compared to control mice (Figure 4E). The control and AAA animals showed a similar pattern of CT enhancement in the other tissues and organs examined (Figure S4).

To confirm our observations in a second murine model, AAA was induced by topical application of elastase to infra-renal abdominal aorta of C57Bl/6J mice treated with β-aminopropionitrile (BAPN). This led to considerable aortic remodeling over a period of 4 to 6 weeks (Figure S5A). In these mice, the dilation of infra-renal abdominal aorta was readily detectable on 5-minute post-contrast CT images (Figure S5B). As in the Ang II-induced AAA model, delayed CT images 24 h post-contrast showed considerable nanoparticle uptake along the aneurysm wall. Quantitative analysis of pre- and post-contrast images showed a significant increase in the Exitron signal volume and maximal radiodensity at 24 h (P < 0.01 vs. pre-injection for both, Figure S3B-C).

To investigate the feasibility of serial imaging, weekly CT imaging was performed in a subgroup of Ang II-infused *Apoe*^-/-^ mice. In the period of 6 days between consecutive injections, the signal persisted in organs rich in MPS cells with a mean radiodensity reduction of 14.7 ± 18.1% for the liver and 0.8 ± 12.0% for the spleen. In AAA, the maximal radiodensity decreased by 10.0 ± 19.5% over the same period of time, while blood and other tissue showed a return to pre-contrast radiodensity levels (Figure S6). As such, successive administration of the contrast agent led to accumulation of nanoparticles in the liver, spleen and AAA.

**Figure 5:**
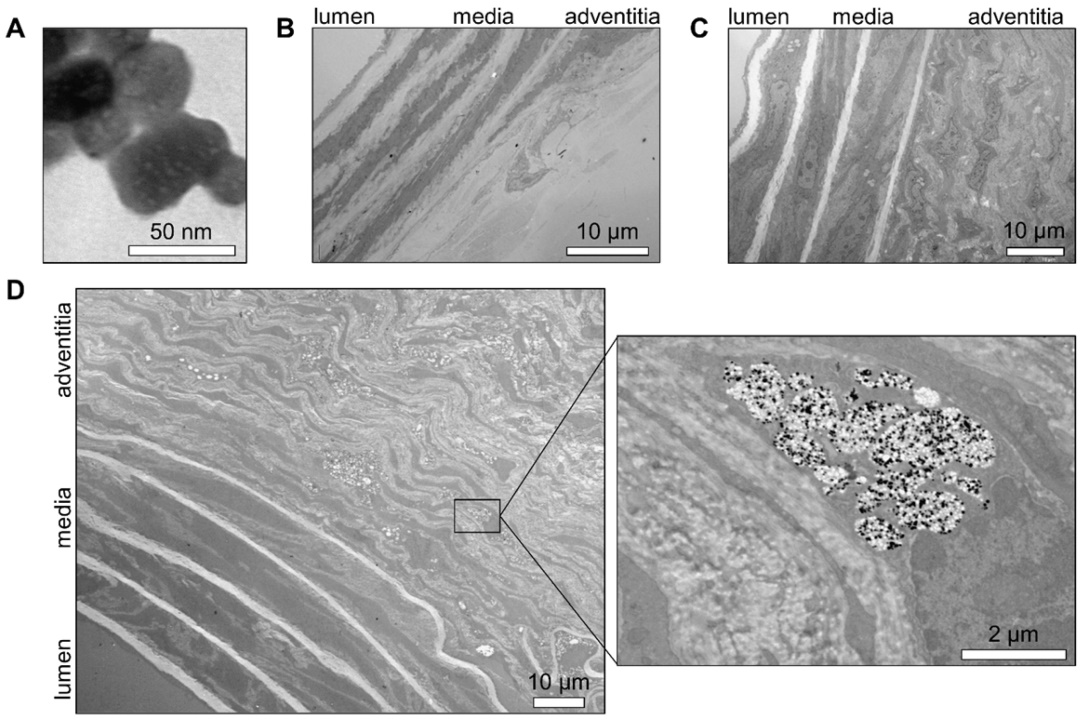
Transmission electron microscopy evaluation of aortic nanoparticles uptake. Representative transmission electron microscopy images of (**A**) Exitron nano 12000 nanoparticles; (**B**) normal murine abdominal aorta isolated 24 h after Exitron nano 12000 administration; (**C**) Ang II-induced AAA tissue from a mouse not injected with contrast agent; (**D**) AAA tissue isolated 24 h after the last injection of Exitron nano 12000, where Exitron nanoparticles can be seen as electron dense particles within adventitial cells.

The location of nanoparticles within the AAA wall was addressed by transmission electron microscopy (TEM). First, we determined the appearance of individual nanoparticles of Exitron on TEM as electron-dense particles (Figure 5A). This pattern was quite unique, as AAA tissue sections from an animal without contrast agent injection did not display any element on TEM that could be mistakenly identified as nanoparticles (Figure 5B). The tissue sections of non-aneurysmal abdominal aorta collected 24 h after the injection of Exitron did not show any nanoparticle within the aortic wall (Figure 5C). This contrasted with AAA tissue sections from an animal with high Exitron uptake after 4 weeks of follow-up by serial imaging that showed aortic wall accumulation of nanoparticles, mainly in the remodeled adventitia (Figure 5D). Higher TEM magnification revealed that the nanoparticle clustered within single-membraned intra-cellular compartments of adventitial cells, either as electron-dense particles or empty areas, interpreted as holes left behind by nanoparticles dislodged during tissue processing (Figure 5D).

### Correlates and predictive value of nanoparticle uptake in AAA

**Figure 6:**
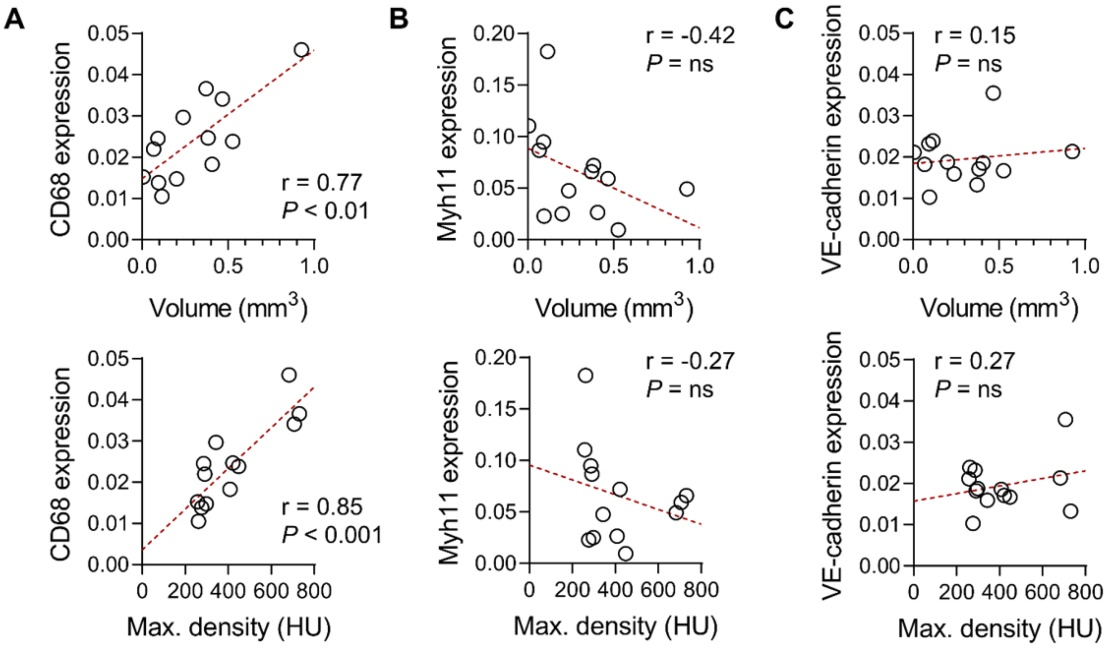
Correlates of Exitron nano 1200 signal in Ang II-induced AAA. Correlation between the suprarenal abdominal aorta CT signal in Ang II-infused *Apoe^-/-^* mice, expressed as enhancement volume (top panel) or maximal radiodensity (bottom panels), with CD68 (**A**), Myh11 (**B**) or VE-cadherin (**C**) gene expression (n = 13). The Pearson correlation coefficient was used to assess the association between variables. HU: Hounsfield Units.

Gene expression analysis of abdominal aortic tissues from Ang II-infused *Apoe*^-/-^ mice showed a significant correlation between CD68 gene expression and aortic wall nanoparticle uptake, quantified as either the volume or maximal radiodensity of the signal (r = 0.77, *P* < 0.01 and r = 0.85, *P* < 0.001, respectively, Figure 6A). However, there was no significant correlation between the smooth muscle cell marker, Myh11, or the endothelial cell marker, VE-cadherin gene expression, and the aortic wall nanoparticle signal (Figure 6B-C).

**Figure 7:**
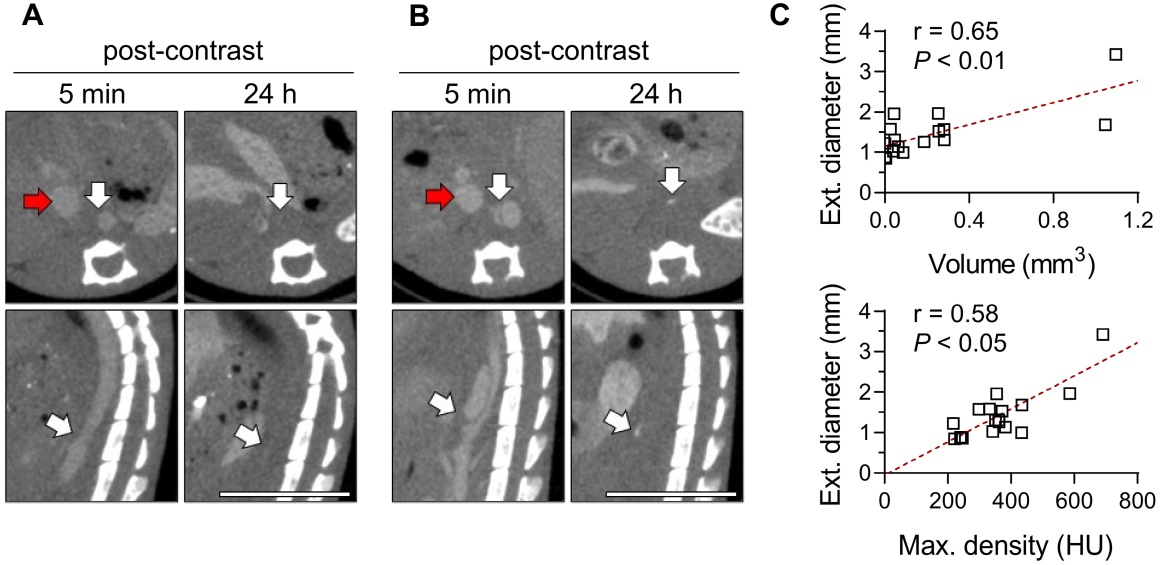
Exitron nano 12000 imaging and AAA outcome. (**A**, **B**) Examples of CT images of mice at days 5-8 of Ang II infusion, representing animals with low (**A**, volume: 0.0 mm^3^, max. density 240 HU) and high (**B**, volume: 1.1 mm^3^, max. density 691 HU) aortic nanoparticle signal. The maximum external supra-renal aortic diameter measured in these animals at 4 weeks were 0.9 mm and 3.4 mm, respectively. White arrows: AAA or moderately remodeled abdominal aorta; red arrows: inferior vena cava. Scale bar: 1 cm. CT scale: -750 to 1250 HU. (**C**) Correlation between nanoparticle enhancement volume (top panel) or maximal radiodensity (bottom panel) at day 5-8 and maximal external aortic diameter at 4 weeks (n = 17), assessed using Spearman's rank-order correlation. HU: Hounsfield Units.

Given its association with macrophages, we investigated the predictive value of the Exitron signal in AAA by evaluating the correlation between the CT signal on images acquired on day 5-8 of Ang II (Figure 7A-B) infusion and AAA outcome (survival and maximal external diameter) after 4 weeks. The Exitron signal at week 1 correlated with maximal aortic diameter at week 4 (Figure 7C). One case of AAA rupture (6%, 1/18) was observed in an animal with high Exitron uptake.

## Discussion

This study established the feasibility of CT-based molecular imaging of vessel wall inflammation in AAA. The phagocytic activity of macrophages was leveraged to target inflammation using a nanoparticle CT contrast agent. The nanoparticles accumulated within the macrophages of the remodeled adventitia creating a CT signal that correlated with the macrophage marker, CD68 expression. Importantly, the CT signal on images obtained within one week of AAA induction predicted the AAA outcome at 4 weeks, directly linking the extent of inflammation to AAA progression.

Vessel wall inflammation involves cells from adaptive and innate immunity, including T and B cells, neutrophils, mast cells and macrophages. Activated immune cells generate proinflammatory cytokines, radical oxygen species and proteolytic enzymes, which lead to the degradation of the extracellular matrix 5. Those stimuli are also involved in vascular smooth muscle phenotypic switch and apoptosis 20, 21. Macrophages, primarily originating from circulating monocytes, are key components of this inflammatory process, and are directly involved in a positive feedback loop that sustains chronic inflammation. While monocyte depletion and attenuation of proinflammatory signaling and chemotaxis ameliorate experimental AAA, these interventions are also associated with major effects on other aspects of animal biology, which potentially confound scientific observations 5, 6. The observed relationship in an intact animal between vessel wall phagocytic activity assessed by CT, and AAA progression in the absence of any other intervention, directly implicates macrophages, the main phagocytic cells in the vessel wall, in AAA evolution.

Classically, evaluation of inflammation requires access to surgical or post-mortem tissue for histological examination. This is incompatible with longitudinal studies and serial analyses, and may have contributed to the incomplete understanding of AAA pathophysiology and absence of specific drugs to treat AAA. This major challenge may be addressed by access to non-invasive imaging tools to evaluate vessel wall inflammation. *In vivo*, inflammation can be detected through several approaches, including the detection of non-specific structural changes, leukocyte trafficking, inflammatory cell metabolic and phagocytic activities, and molecular imaging of inflammatory mediators 22. Many of these approaches rely on nuclear imaging that while highly sensitive, is limited with regards to spatial resolution. In AAA, imaging of vessel wall inflammation has mainly relied on the detection of metabolic activity by ^18^F-fluorodeoxyglucose (FDG) positron emission tomography (PET) imaging, and phagocytic activity by USPIO-enhanced MRI 10. However, the FDG signal is not specific for inflammation 23, and MRI is challenging, costly, and difficult to standardize, especially for pre-clinical studies 24.

CT is a versatile and accessible technology for preclinical and clinical applications with high spatial resolution and low cost. With the use of contrast agents, CT angiography enables high resolution anatomical imaging of vascular structures such as murine AAA 25. Beyond imaging of vascular anatomy, the CT's established calibration and quantitative analytical methodology make it an excellent imaging modality to assess tissue biology, including the presence of inflammation. However, the methods for CT imaging of vascular inflammation developed so far show only limited applicability, and there is no report of CT imaging of vessel wall inflammation in AAA. For instance, injecting gold nanoparticle-labeled monocytes to atherosclerotic mice led to their accumulation in atherosclerosis with ~ 15 HU CT enhancement 26. While cell tracking is of interest in certain preclinical studies, such an approach provides limited information about inflammation and the modest enhancement observed is difficult to distinguish from the noise 26, 27. A similar modest increase in radiodensity (~15 HU) was achieved using an iodine-based polymeric nanoparticle formulation, N1177 for CT imaging of mouse and rabbit atherosclerosis 28, 29. Another study using liposomal-iodine nanoparticles showed targeting to murine atherosclerosis by dual-energy CT to account for plaque calcification, however, the lack of calibration prevented quantitative analysis of tissue enhancement 30. Perhaps because of this modest enhancement in animal models, none of these studies have provided any functional information on vessel wall inflammation. In our study, the magnitude of CT enhancement in the hundreds of HU, which led to unambiguous detection of vessel wall phagocytic activity, uniquely enabled us to link the Exitron signal on early images to AAA outcome. With a simple methodology and using a widely available imaging modality, this imaging method can be easily used for a variety of preclinical applications. Further studies, including interventions to modulate tissue inflammation 3, are needed to establish the dynamic range of this method.

Exitron nano 12000 is calibrated to yield a radiodensity of 12000 HU for the undiluted product, equivalent to 600 mg of iodine per kg of body weight at the dose used in this study 31. Nanoparticle characterization confirmed the presence of a barium sulfate core with a mean particle core diameter of 79 nm. While phagocytosis is typically described for larger particle sizes 32, nanoparticles of a similar size or even smaller particles, such as USPIO may be taken up by phagocytosis 33, 34. The physiochemical properties of nanoparticles and their biological behavior *in vivo* are strongly influenced by the adsorption of biomolecules, the formation of a protein corona, and potentially aggregation 35. Accordingly, the professional phagocytes are involved in the clearance of a wide range of nanomaterial 36. The biodistribution pattern of Exitron nano 12000 strongly suggests phagocytic uptake by MPS cells, which is consistent with our TEM analysis of AAA tissue showing the accumulation of nanoparticles within an intra-cellular single-membraned compartment of cells identified as macrophages, the major inflammatory cell type present in the remodeled adventitia in this animal model 37. *In vivo* accumulation of nanoparticles in AAA correlates with CD68 (macrosialin) gene expression. Typically recognized as a macrophage marker, CD68 is a member of the lysosomal-associated membrane proteins and indicative of the cell capacity for phagocytosis 38.

Macrophage polarization influences the phagocytic capability in a substrate-dependent manner 39, although the exact nature of macrophage polarization in our animal models remains to be determined 6. We show that classical polarization of RAW 264.7 macrophages reduces Exitron nano 12000 uptake *in vitro*. A similar reduction in USPIO uptake in classically polarized macrophages has been reported 40, 41. While potentially providing valuable insights, caution is mandated when extrapolating the results from *in vitro* studies to *in vivo* systems 36, 42. This issue is also relevant to Exitron uptake by non-professional phagocytes observed *in vitro*. The relevance of these *in vitro* observations to Exitron uptake *in vivo* and the molecular mechanisms involved in nanoparticle uptake remains an interesting area of future research.

Exitron nano 12000 use is restricted to preclinical applications. As such, the imaging method described in this study is not directly transposable to humans. Toxicity is a common concern for many nanoparticle contrast agents 43-45. While we did not observe any immediate toxicity or an effect on macrophage polarization markers, the accumulation of Exitron nano 12000 in MPS cells over time may suggest long term toxicity, which is beyond the scope of this study. Nonetheless, the work presented here opens an avenue for the development of new clinical contrast agents for CT-based imaging of inflammation in AAA, for which proper evaluation of the toxicological profile would be mandated.

While we observed little vessel wall calcification in our animal models, calcification does occur in aneurysm (and other vascular pathologies) and may interfere with image interpretation. Recent advances in CT technology, including the introduction of dual energy CT scanners 46, should address this limitation of CT imaging and enable the distinction between vessel wall inflammation and calcification. The CT studies in this study were acquired with 50 keV. Given the barium's k-edge of 37 keV, multi-energy CT imaging could further improve the image quality and quantitative accuracy through material decomposition.

In conclusion, this study established the feasibility of CT-based molecular imaging of phagocytic activity in preclinical models of AAA and linked the inflammatory signal on early time point images to AAA outcome. This readily available technology overcomes an important barrier to cross-sectional, longitudinal and outcome studies, not only in AAA, but also in other cardiovascular pathologies. The robustness of the CT signal *in vivo* is a major advantage of this approach, and facilitates the evaluation of modulatory interventions, important for vascular biology research, and drug discovery and development.

## Methods

### *In vitro* characterization of CT contrast agents

Four commercial nanoparticle CT contrast agents with distinct structural compositions were evaluated: Fenestra LC (MediLumine Inc.), Mvivo Au (MediLumine Inc.), Mvivo BIS (MediLumine Inc.) and Exitron nano 12000 (Miltenyi Biotec). The stock solutions and serial dilutions of each contrast agent were imaged by CT and their radiodensity (radiopacity) was quantified (see below). The selected contrast agent, Exitron, was further characterized. SEM, EDS and Raman spectroscopy were performed on air dried specimens of diluted contrast agent, prepared on copper TEM grids coated with a Lacey carbon support film (Electron Microscopy Sciences). The specimens were imaged by SEM (SU8230 UHR Cold Field Emission SEM microscope, Hitachi) and the nanoparticle size was measured using ImageJ software (imagej.net). High spatial resolution EDS was carried out to map the elemental composition of the nanoparticles during SEM acquisition (XFlash 5060FQ Annular EDS detector, Burker). Raman spectroscopy was performed with an excitation wavelength of 532 nm (LabRam HR800 Raman spectrometer, Horiba). A lyophilized sample of the contrast agent was used for FTIR spectroscopy in the attenuated total reflectance mode (Cary 660 FTIR instrument, Agilent). Raman and FTIR spectra were compared and matched with the RRUFF database (ID: R050342) 47. XPS was performed in an ultrahigh vacuum environment (10^-9^ mbar) using the Al Kα anode (1486.6 eV) at a pass energy of 40 eV (VersaProbe II Scanning XPS Microprobe, PHI), and processed using the FsemPHI MultiPak software. A water diluted sample of the contrast agent (1:750) was used to determine the zeta potential (ZetaPALS, NanoBrook).

### Nanoparticle uptake assay and *in vitro* evaluation

RAW 264.7 macrophages (ATCC TIB-71), bEnd.3 endothelial cells (ATCC CRL-2299) and MOVAS smooth muscle cells (ATCC CRL-2797) were cultured in Dulbecco's Modified Eagle's Medium supplemented with fetal bovine serum 10%, L-glutamine 10 mM, penicillin 100 U/mL and streptomycin 0.1 mg/mL (or Geneticin 0.2 mg/mL for MOVAS) at 37 °C with 5% CO_2_ and saturated humidity. The cells were plated in 60-mm dishes (or 100-mm dishes for b.End.3 cells) 1 day before the nanoparticle uptake assay. RAW 264.7 cells were polarized either with 0.1 µg/mL LPS and 0.1 µg/mL IFNγ, or 0.1 µg/mL IL-4 for 24 h and cell polarization was verified by quantitative RT-PCR. Nanoparticle contrast agents diluted in fresh media at dilutions yielding the same radiodensity (1:128, 1:150, 1:153, and 1:23 for Mvivo BIS, Exitron and Mvivo Au, and Fenestra LC respectively, corresponding to 20% of the initial Exitron concentration in blood) were incubated for 4 h at 37 °C or 4 °C. The cells were washed to remove any non-internalized contrast agent, trypsinized, transferred to 0.2 mL tubes and centrifuged for 2 min. The supernatant was removed and the tubes with cell pellets were imaged by CT and the radiodensity of the cell pellet was quantified (see below for details). Additional RAW 264.7 cells plated in 35-mm culture dishes were exposed to Exitron (1:150) for 1 h, 18 h and 48 h and cell polarization was assessed by quantitative RT-PCR.

### Animal studies

Wild-type C57BL/6J and apolipoprotein E-deficient (*Apoe^-/-^*) mice, originally purchased from the Jackson Laboratories, were used in this study. For biodistribution studies, male C57BL/6J mice (n = 5) were imaged by CT prior to the injection of the contrast agent and serial CT scans were performed at 5 min, 1 h, 2 h, 4 h, 6 h, 8 h, 12 h and 24 h after the injection of 50 μL per 25 g of bw of Exitron. For the first model of AAA, male *Apoe^-/-^* mice were implanted with an osmotic minipump (model 2004, Alzet) delivering recombinant human angiotensin II at 1000 ng/min/kg for up to 4 weeks to induce aneurysm in the supra-renal abdominal aorta 48. In a group of *Apoe^-/-^* mice (n = 13), animals at 2 or 4 weeks of Ang II infusion underwent a pre-contrast CT acquisition, followed by the injection of Exitron (50 μL/25 g of bw) and post-contrast CT scans at ~ 5 min p.i. and 24 h p.i. prior to euthanasia and tissue harvesting. For longitudinal studies, a group of animals (n = 18) underwent Exitron administration at 4-7 days of Ang II infusion with 3 CT acquisitions prior to, and at 5 min and 24h post-contrast administration. These animals were followed for 3 more weeks and tissue samples were collected from surviving animals. A subset of these animals (10/18) underwent weekly 3-CT image acquisition with additional molecular imaging studies (6/10) at 2-3 weeks of Ang II infusion. Additional animals were used for evaluation by electron microscopy (n = 2). The aortic diameter of saline-infused animals was from previously published data 49. For the second model of AAA, C57BL/6J mice (n = 10) were used to generate infra-renal aortic aneurysm , as described 50. Briefly, animals were treated with β-aminopropionitrile (BAPN, Sigma) 0.2% in drinking water. Two days after the start of BAPN administration, the animals underwent surgery. The infra-renal abdominal aorta was exposed and porcine pancreatic elastase (6.67 mg/mL, 10 U/mg, MP Biomedical) was topically applied for 5 min. At 4-6 weeks after surgery, the animals underwent the same 3 CT scans acquisition protocol, followed by animal euthanasia and tissue harvesting. A chart summarizing the animal experiments is included in the supporting material (Figure S7). All procedures were performed following the regulations of Yale University and VA Connecticut Healthcare System's Animal Care and Use Committees.

### Computed tomography imaging

CT scanning was performed on a small animal-dedicated SPECT/CT scanner (U-SPECT^4^CT, MILabs) using one bed position at the maximal field of view (total body mode) with 180 projections (0.5° step angle, single projection per step) of 40 ms with a beam energy of 50 kV and 0.43 mA. The camera was calibrated with those parameters to provide the radiodensity in Hounsfield units (HU). Images were reconstructed at 100 μm of isotropic voxel size with Gaussian filter at 150 μm full-width at half-maximum using the manufacturer's software.

### Image analysis

CT Images were quantified using 3D Slicer software (www.slicer.org). The cell pellet radiodensity was measured in volumes of interest (VOI) drawn by visual thresholding. The average radiodensity in various tissues (blood, liver, spleen, lung, muscle and kidney) was measured using VOI placed based on anatomical landmarks. Areas of nanoparticle uptake in AAA were defined as those with a radiodensity ≥ 200 HU along the abdominal aorta. This threshold was determined empirically (Figure S8). The maximal radiodensity in segments with a radiodensity ≥ 200 HU was recorded. Volume thresholding was applied using a custom script written in MATLAB R2019b. Clusters of voxels were identified and labelled as individual objects using connected component analysis. The voxel connectivity was set to 26 (6 faces, 12 edges and 8 corners of a voxel). The criterion for an object to be classified as true signal was defined as ≥ 5 voxels. Objects which did not meet the criterion were considered as noise and excluded, and the volume of the remaining objects (in mm^3^) was recorded. The intra- and inter-observer reproducibility of the analysis was assessed in 2 distinct sets of images, demonstrating excellent intraclass correlation coefficients (≥ 0.94) for all analyses.

### Tissue processing

The animals euthanized at 24 h after a single injection of contrast agent were perfused with heparinized saline solution. The aorta was carefully harvested, placed in Optimal Cutting Temperature compound (Tissue Tek), snap frozen, and processed using a cryostat (CM1850, Leica). Serial sections were collected at regular intervals and used for morphometry and immunofluorescence staining. The tissue between these sections was collected for RNA extraction. Other animals were perfused with 4% paraformaldehyde (PFA), their aortae harvested and kept in 4% PFA for 48 h prior to the transfer to OCT and morphometry analysis. Additionally, a small segment of aneurysm tissue (~ 1 mm long) at the site of intense uptake on CT images was collected with the help of anatomical landmarks from an animal with high nanoparticle uptake. This segment of aorta was processed for TEM imaging, as described 51. Briefly, the tissue sample were fixed with 2.5% glutaraldehyde/2% PFA in 0.1 M sodium cacodylate buffer, post-fixed in 1% osmium tetroxide, stained *en bloc* in 2% aqueous uranyl acetate, dehydrated and embedded in resin, sectioned at 60 nm (UltraCut UC7, Leica) and collected on carbon/formvar‐coated grids for electron microscopy imaging. The AAA tissue from an *Apoe^-/-^* mouse not injected with Exitron and non-aneurysmal aortic tissue from an *Apoe^-/-^* mouse at 24 h after the injection of Exitron were processed similarly and used as control.

### Morphometry, immunostaining and transmission electron microscopy

For morphometry, images of serial tissue sections of the abdominal aorta were acquired using a microscope (Eclipse E400, Nikon) and the maximal external diameter was measured using ImageJ/Fiji software (imagej.net). For immunostaining, the tissue sections were incubated with a primary antibody (CD68: 1:100, MA5-16674, Invitrogen; α-smooth muscle actin: 1:100, A2547, Sigma-Aldrich; CD31: 1:100, 14-0311-82, Invitrogen) overnight at 4 °C, followed by incubation with the appropriate fluorochrome-conjugated secondary antibody (1:200, A-11007 or A-11005, Invitrogen). The images were acquired using a fluorescence microscope (DMi8, Leica). TEM images were acquired on a transmission electron microscope at 80 kV (Biotwin, FEI Tecnai) with Morada CCD and iTEM (Olympus) software.

### Quantitative RT-PCR

Total RNA was isolated from cells or the abdominal aortic tissue (RNeasy Mini Kit, Qiagen) and reverse transcribed (QuantiTect Reverse Transcription Kit, Qiagen). RT-PCR was performed on a Real-Time PCR System (Applied Biosystems) using the following TaqMan primers and probe sets (β-actin: Mn00607939_s1, Nos2: Mm00440502_m1, Mrc1: Mm01329359_m1, CD68: Mm03047343_m1, Myh11: Mm00443013_m1, VE-cadherin: Mm00486938_m1, Thermo Fisher Scientific). Gene expression data are presented as 2^-ΔCT^, normalized to β-actin.

### Statistics

Continuous variables are presented as mean ± standard deviation. The normality of distribution was assessed using the Shapiro & Wilk normality test. For unpaired variables, the differences between the means of two or multiple variables were assessed by unpaired t-test and one-way ANOVA with Tukey's correction, respectively for normally distributed variables, and Mann-Whitney test and Dunn's multiple comparison test, respectively for non-normally distributed variables. For repeated measures of normally distributed variables, the differences between the means was assessed by one-way ANOVA with Geisser-Greenhouse and Tukey 's corrections. For analysis of paired variable, the differences between the means were evaluated using paired t-test or Wilcoxon matched-pairs signed rank test for normally and not normally distributed variables, respectively. Kaplan-Meier survival curves were compared using Log-rank (Mantel-Cox) test. The association between two normally and non-normally distributed variables were evaluated using Pearson's and Spearman's correlation coefficients, respectively. All statistical analyses were performed using Prism 8 (GraphPad).

## Supplementary Material

Supplementary figures.

## Abbreviations

AAA: abdominal aortic aneurysm
Ang II: angiotensin II
Apoe: apolipoprotein E
BAPN: β-aminopropionitrile
BW: body weight
CT: computed tomography
EDS: energy-dispersive X-ray spectroscopy
FDG: 18F-fluorodeoxyglucose
FTIR: Fourier-transform infrared spectroscopy
HU: Hounsfield units
IL-4: interleukin-4
INFγ: interferon gamma
LPS: lipopolysaccharide
MPS: mononuclear phagocytic system
MRI: magnetic resonance imaging
PET: positron emission tomography
SEM: scanning electron microscopy
TEM: transmission electron microscopy
USPIO: ultrasmall superparamagnetic iron oxide particles
XPS: X-ray photoelectron spectroscopy
