# Supplementary material for: Computed tomography imaging of macrophage phagocytic activity in abdominal aortic aneurysm
Source: Theranostics. 2021 Apr 3;11(12):5876–88. doi: 10.7150/thno.55106 (PMC8058712; doi:10.7150/thno.55106)
Supplement: Supplementary file 1 — Supplementary figures. [file thnov11p5876s1.pdf]

Computed tomography imaging of macrophage phagocytic activity in abdominal aortic aneurysm

Jakub Toczek[1,2], Parnaz Boodagh[1,2], Nowshin Sanzida[1,2], Mean Ghim[1,2], Mani Salarian[1,2], Kiran Gona[1,2], Gunjan Kukreja[1,2], Saranya Rajendran[1,2], Linyan Wei[1,2], Jinah Han[1,2], Jiasheng Zhang[1,2], Jae-Joon Jung[1,2], Morven Graham[3], Xinran Liu[3] and Mehran M. Sadeghi[1,2,*]

[1] Cardiovascular Molecular Imaging Laboratory, Section of Cardiovascular Medicine and Yale Cardiovascular Research Center, Yale University School of Medicine, New Haven, CT (USA)

[2] Veterans Affairs Connecticut Healthcare System, West Haven, CT (USA)

[3] CCMI Electron Microscopy Core Facility, Yale University School of Medicine, New Haven, CT (USA)

*To whom correspondence should be addressed: Mehran M. Sadeghi, M.D., Professor of Medicine (Cardiology), Yale School of Medicine, 300 George Street, Room 770G, New Haven, CT 06511, Phone: 203-737 6954, Fax: 203-937 3884, Email: mehran.sadeghi@yale.edu

Supplementary Material

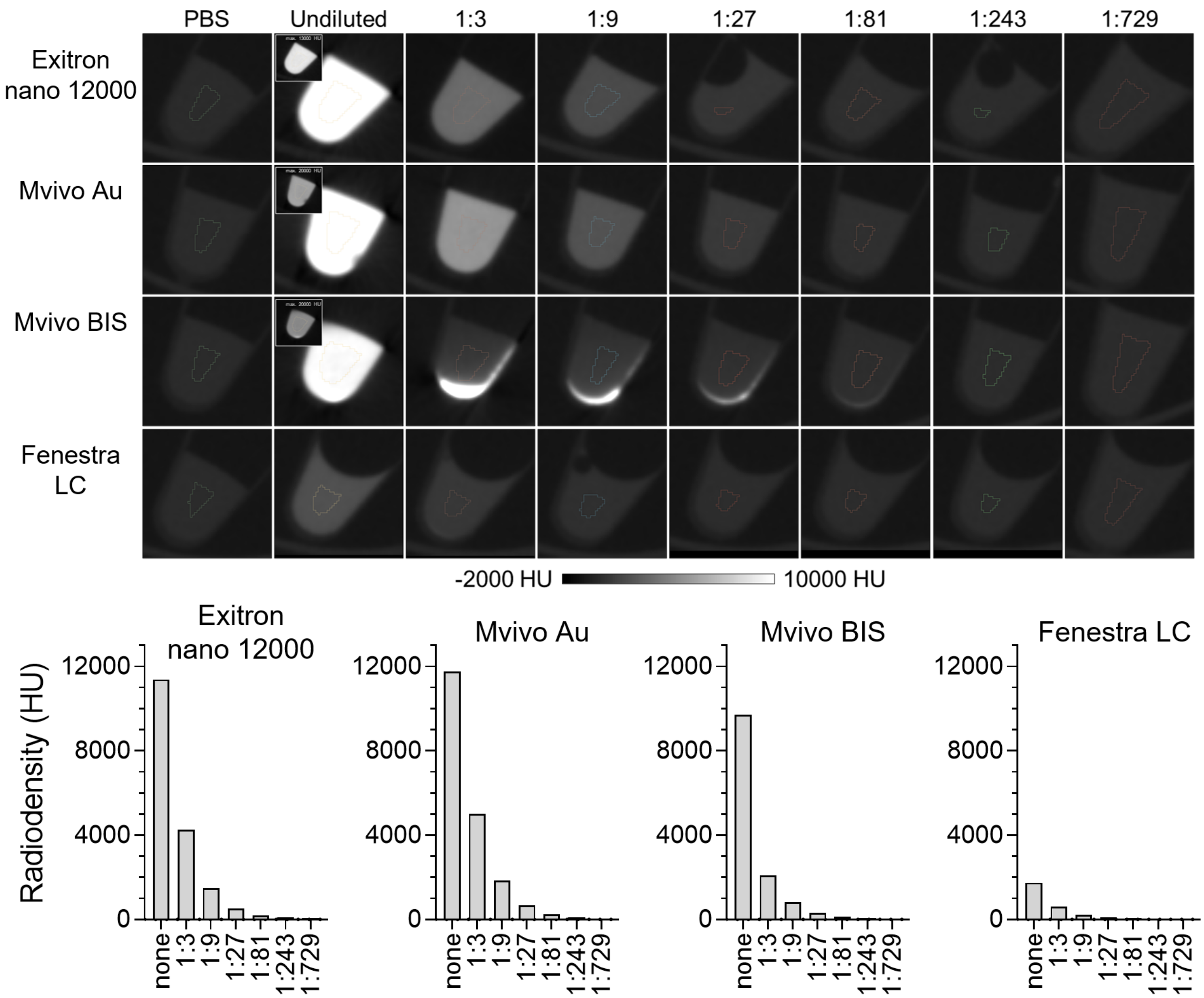

Figure S1

Radiodensity of CT contrast agents. CT images and radiodensity quantification of serial dilutions of selected nanoparticle contrast agents. PBS: Phosphate buffered saline, HU: Hounsfield units.

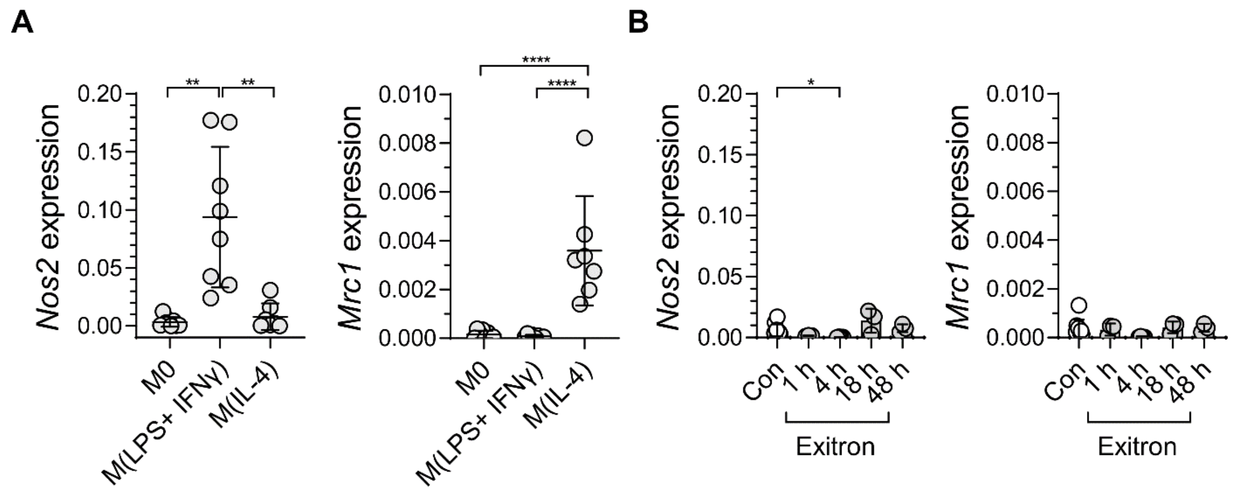

Figure S2

RAW 264.3 macrophage polarization and impact of Exitron nano 12000 exposure on polarization. (**A**) Gene expression analysis of RAW 264.7 cell polarization markers *Nos2* and *Mrc1* in untreated cells (M0), after LPS and IFNγ stimulation [M(LPS+ IFNγ)] and IL-4 stimulation [M(IL-4)] (n = 7–8). Statistical significance was assessed by Kruskal-Wallis test with Dunn's corrections and one-way ANOVA for *Nos2* and *Mrc1* gene expression, respectively. (**B**) Evaluation of polarization markers *Nos2* and *Mrc1* in RAW 264.7 cell without and after 1 h, 4 h, 18 h and 48 h of Exitron nano 12000 exposure (n = 3–4 for Exitron, n = 10 for Control). Statistical significance was assessed by Dunn's multiple comparison test.

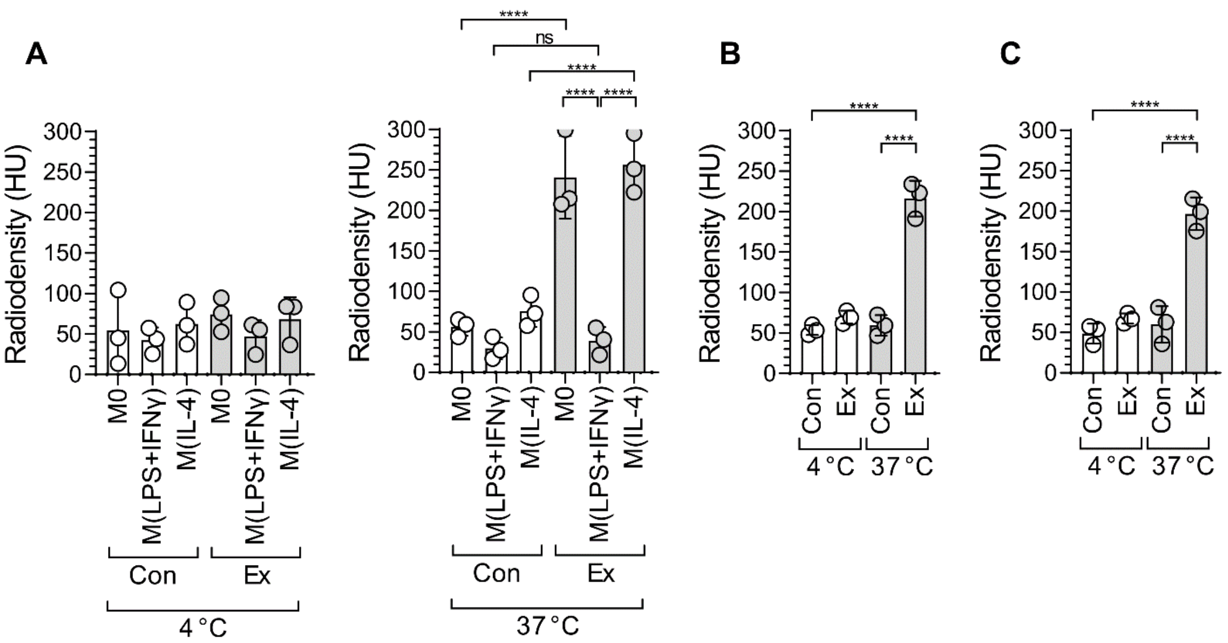

Figure S3

In vitro evaluation of Exitron nano 12000 uptake in unpolarized (M0) and polarized [M(LPS+IFNγ) and M(IL-4)] RAW 264.7 macrophages (**A**), b.END3 endothelial cells (**B**) and MOVAS aortic smooth muscle cells (**C**) at 4 and 37 °C (n = 3). Statistical significance was assessed by Dunn's multiple comparison test (left panel in A) or one-way ANOVA (all other panels). Ex: Exitron nano 12000; HU: Hounsfield units.

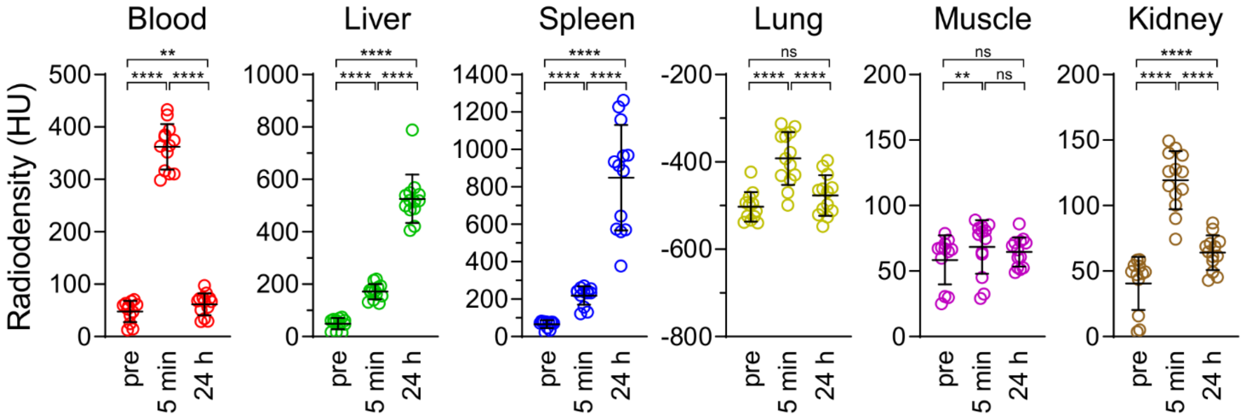

Figure S4

Exitron nano 12000 biodistribution in Ang II-infused *Apoe*$^{-/-}$ mice. Average tissue radiodensity quantified on in vivo CT images of *Apoe*$^{-/-}$ mice pre-, and at 5 min and 24 h post-Exitron administration. Statistical significance was assessed by one-way ANOVA with Tukey's correction (n=13). ** $P < 0.01$, *** $P < 0.001$, **** $P < 0.0001$, ns: not significant. HU: Hounsfield Units.

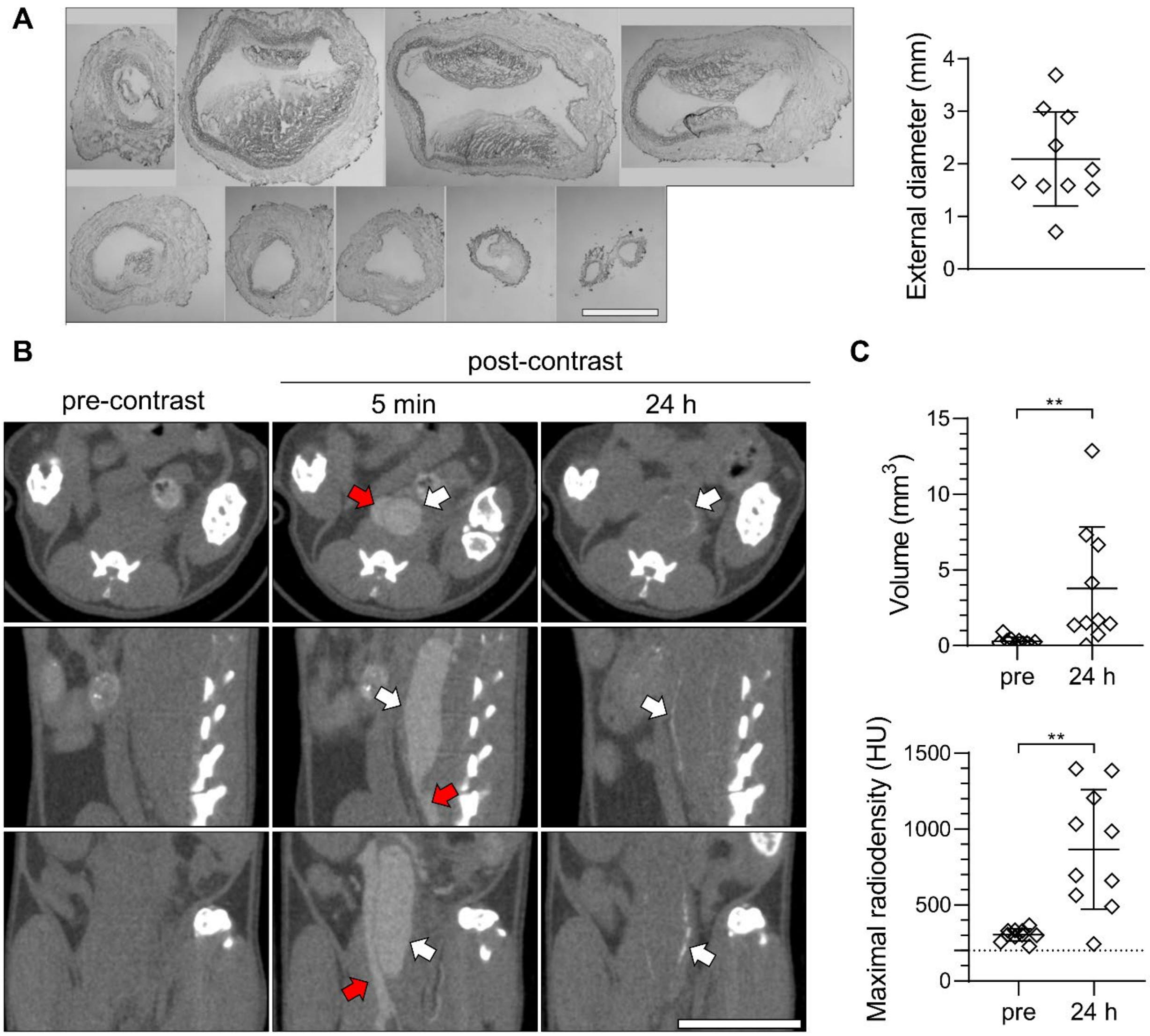

Figure S5

Exitron nano 12000 uptake in topical elastase-induced murine AAA. (**A**) Brightfield images of serial sections of infra-renal abdominal aorta (proximal to distal from left to right) from a C57Bl/6J mouse at 6 weeks after topical elastase application (scale bar: 1 mm), and maximal external diameter of the infra-renal aorta at 4 to 6 weeks after topical elastase application in β-aminopropionitrile-treated C57Bl/6J mice. (**B**) CT images of a representative C57Bl/6J mouse at

4 weeks after topical elastase application pre- (left panels), and at 5 min (middle panels) and 24 h (right panels) post-Exitron nano 12000 administration. White arrows: AAA; red arrows: inferior vena cava. Scale bar: 1 cm. CT scale: -750 to 1250 HU. (**C**) Quantification of the CT signal presented as enhancement volume (top panel) and maximal radiodensity (bottom panel). Statistical analysis was performed using Wilcoxon signed-rank test. ** $P < 0.01$. HU: Hounsfield Units.

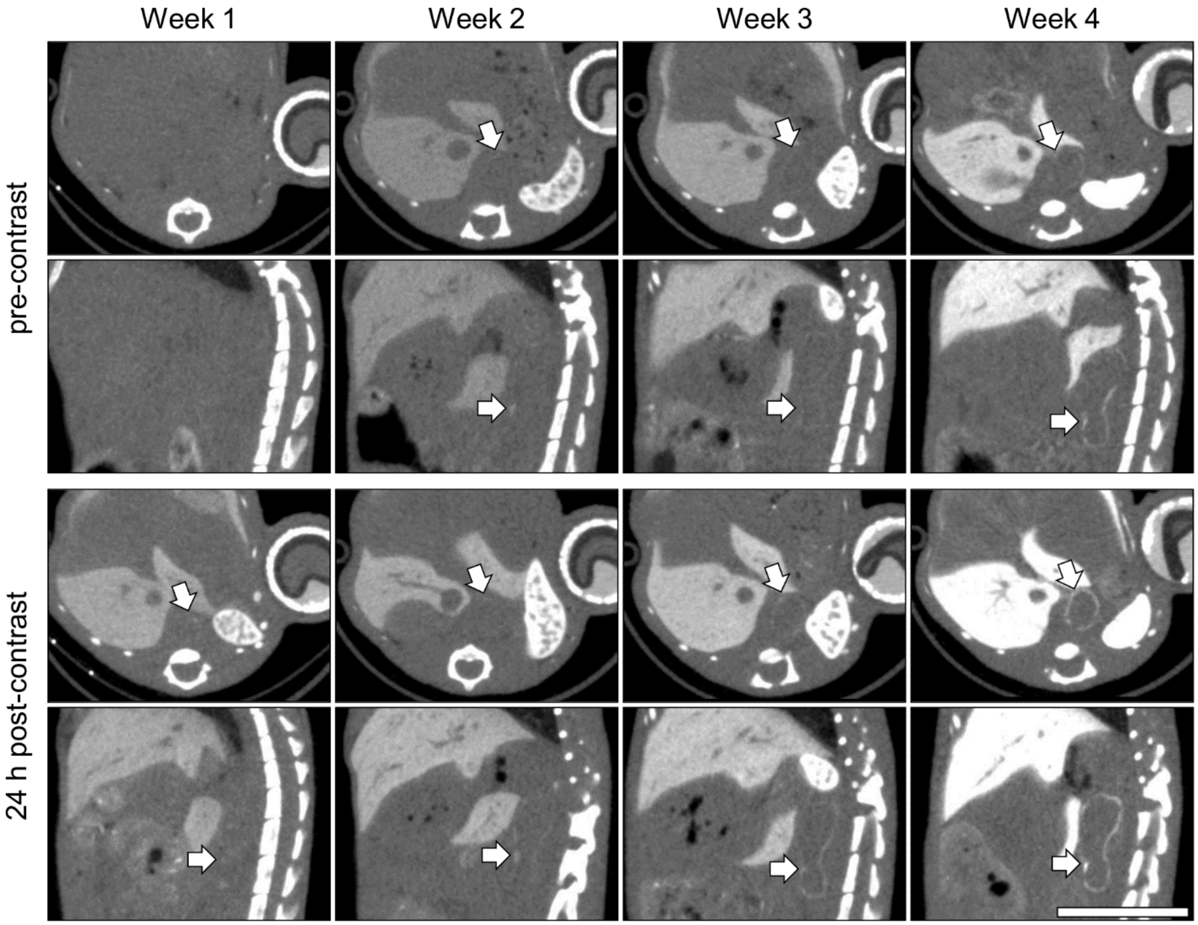

Figure S6

Evolution of CT signal with AAA progression. Serial CT images obtained weekly before (top panels) and 24 h after Exitron nano 12000 administration (bottom panels) from an *Apoe*$^{-/-}$ mouse infused with Ang II for 4 weeks. White arrows: AAA. Scale bar: 1 cm. CT scale: -750 to 1250 Hounsfield Units.

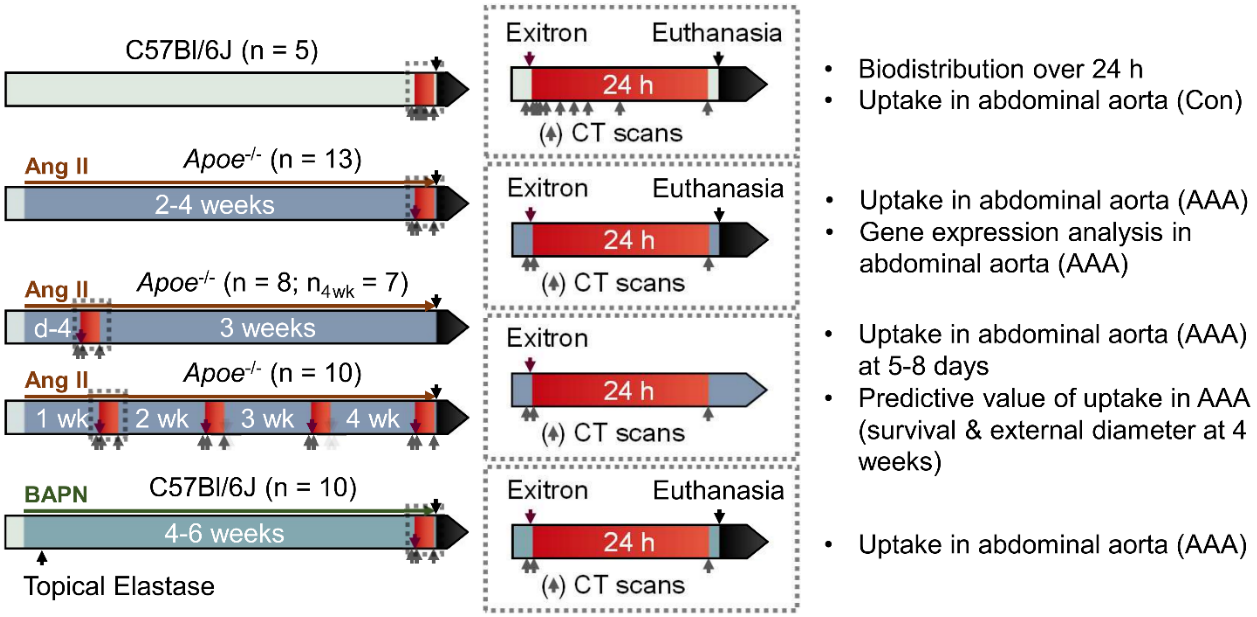

Figure S7

Flow chart of animal studies.

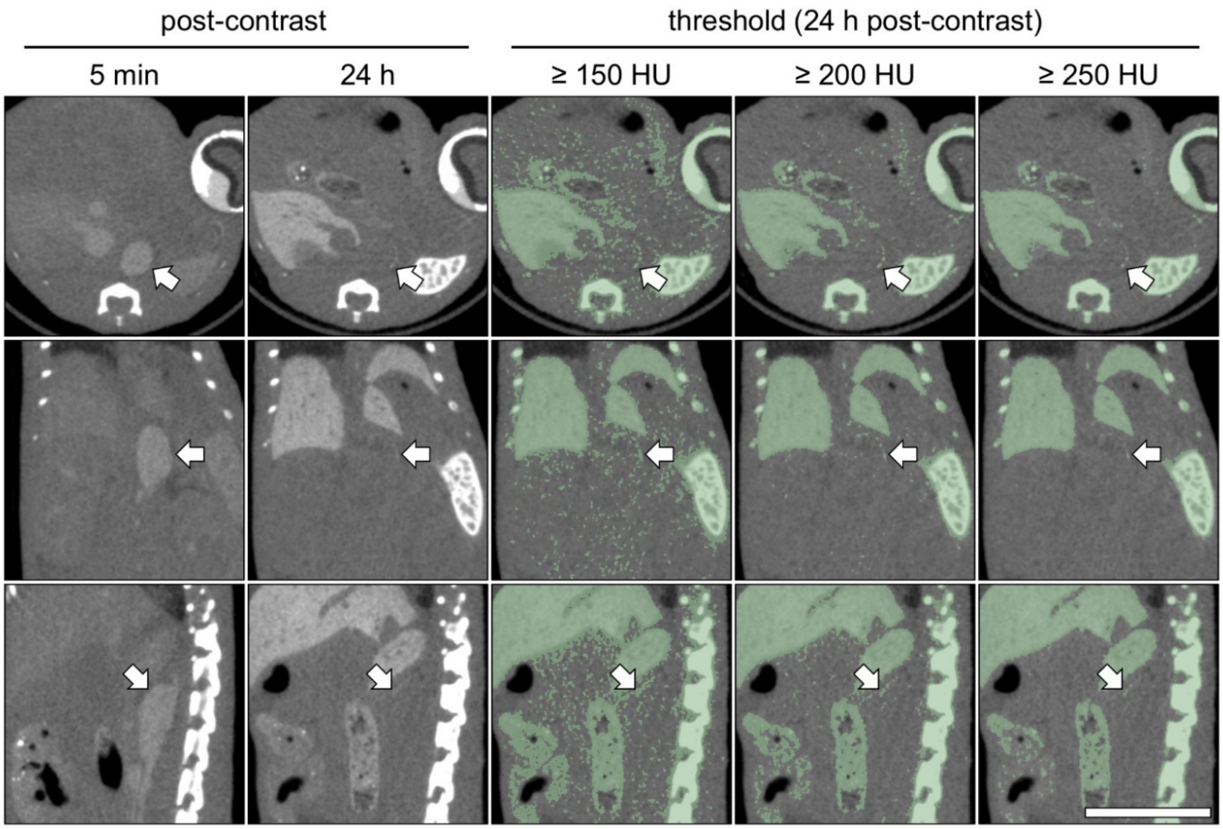

Figure S8

Thresholding of CT images acquired 24 h post-Exitron administration. CT images of a representative Ang II-infused *Apoe*$^{-/-}$ mouse acquired at 5 min and 24 h after Exitron nano 12000 administration (left panels). The pixels with radiodensities ≥ 150 Hounsfield Unit (HU), 200 HU and 250 HU thresholds are colored in green on delayed CT images (right panels). White arrow: AAA. CT scale: -750 to 1250 HU. Scale bar: 1 cm. HU: Hounsfield units.
